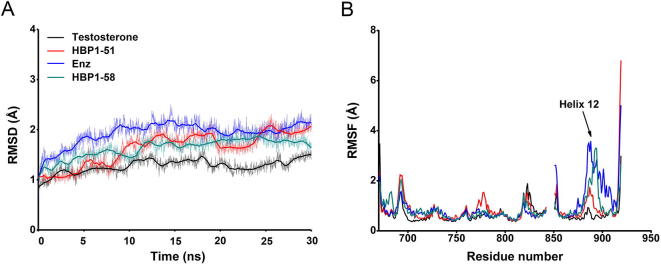# Discovery of Novel Androgen Receptor Ligands by Structure-based Virtual Screening and Bioassays

**DOI:** 10.1016/j.gpb.2018.03.007

**Published:** 2019-01-09

**Authors:** Wenfang Zhou, Mojie Duan, Weitao Fu, Jinping Pang, Qin Tang, Huiyong Sun, Lei Xu, Shan Chang, Dan Li, Tingjun Hou

**Affiliations:** 1 College of Pharmaceutical Sciences, Zhejiang University, Hangzhou 310058, China; 2 State Key Laboratory of Computer Aided Design and Computer Graphics (CAD&GC), Zhejiang University, Hangzhou 310058, China; 3 State Key Laboratory of Magnetic Resonance and Atomic and Molecular Physics, National Center for Magnetic Resonance in Wuhan, Wuhan Institute of Physics and Mathematics, Chinese Academy of Sciences, Wuhan 430071, China; 4 Institute of Bioinformatics and Medical Engineering, School of Electrical and Information Engineering, Jiangsu University of Technology, Changzhou 213001, China

**Keywords:** Androgen receptor, AR ligand, Virtual screening, AR agonist, AR antagonist

## Abstract

**Androgen receptor** (AR) is a ligand-activated transcription factor that plays a pivotal role in the development and progression of many severe diseases such as prostate cancer, muscle atrophy, and osteoporosis. Binding of ligands to AR triggers the conformational changes in AR that may affect the recruitment of coactivators and downstream response of AR signaling pathway. Therefore, **AR ligands** have great potential to treat these diseases. In this study, we searched for novel AR ligands by performing a docking-based **virtual screening** (VS) on the basis of the crystal structure of the AR ligand binding domain (LBD) in complex with its agonist. A total of 58 structurally diverse compounds were selected and subjected to LBD affinity assay, with five of them (HBP1-3, HBP1-17, HBP1-38, HBP1-51, and HBP1-58) exhibiting strong binding to AR-LBD. The IC_50_ values of HBP1-51 and HBP1-58 are 3.96 µM and 4.92 µM, respectively, which are even lower than that of enzalutamide (Enz, IC_50_ = 13.87 µM), a marketed second-generation **AR antagonist**. Further bioactivity assays suggest that HBP1-51 is an **AR agonist**, whereas HBP1-58 is an AR antagonist. In addition, molecular dynamics (MD) simulations and principal components analysis (PCA) were carried out to reveal the binding principle of the newly-identified AR ligands toward AR. Our modeling results indicate that the conformational changes of helix 12 induced by the bindings of antagonist and agonist are visibly different. In summary, the current study provides a highly efficient way to discover novel AR ligands, which could serve as the starting point for development of new therapeutics for AR-related diseases.

## Introduction

Androgen receptor (AR) is a member of the steroid hormone nuclear receptor (NR) superfamily [1]. Stimulation of AR by endogenous androgens, such as testosterone and its metabolite dihydrotestosterone (DHT), influences the expression of a series of downstream genes essential to the growth and development of mammals [2]. Moreover, AR is involved in a number of severe diseases, such as prostate cancer (PCa) [2], muscle atrophy [3], and osteoporosis [4]. PCa is a widespread disease in men and is one of the leading causes for cancer death worldwide. It has been reported that AR gene and protein are both generally overexpressed at late stage of PCa [5], [6]. AR overexpression augments the response of AR signaling pathway to residual androgens, and is one of the main driving forces for the progression into the lethal castration-resistant PCa [5], [6]. In fact, AR antagonists such as the well-known flutamide and bicalutamide (Bic) have been successfully used to treat PCa for years [2].

Other than PCa, muscle atrophy [3] and osteoporosis [4], which commonly occur with advanced ages, result in reduced muscle and bone mass and increased risk of physical disability and fractures, thus adversely influencing patients’ life quality. AR agonists such as testosterone can boost anabolic reactions and are beneficial for the treatment of these two diseases, albeit the detailed mechanism remains unclear [4]. In addition, AR is considered as a potential drug target for breast cancer, ovarian cancer, and pancreatic cancer. Therapies involving AR antagonists in the treatment of these diseases are now being tested in clinical trials at phase I or II (NCT02697032, NCT01974765, and NCT02138383). The versatile therapeutic applications of AR ligands make them hot spot for pharmaceutic studies.

In recent years, computational virtual screening (VS) has been used to identify novel AR antagonists owing to its high efficiency compared with traditional experimental assays. For example, Song and co-workers have identified a novel AR antagonist 6-(3,4-dihydro-1H-isoquinolin-2-yl)-N-(6-methylpyridin-2-yl)nicotinamide (DIMN) through a structure-based VS using the FlexX docking algorithm, which exhibits comparable antagonistic effect with hydroxyflutamide and Bic [7]. Additionally, Li et al. [8] employed an integrated strategy by combining structure- and ligand-based VS involving two docking techniques (*Glide* and eHiTs) and a quantitative structure activity relationship (QSAR) model. They have identified a number of novel AR antagonists with submicromolar activities, therein the best hit VPC-12060 shows anti-AR potency similar to Bic *in vitro*. These studies indicate that combination of computational VS and biological experiments is a powerful way to identify novel AR antagonists. Furthermore, molecular docking has long been used for *in silico* screen of small molecules with high affinity to a target, which would be appropriate for discovering not only AR antagonists but also AR agonists.

Besides antagonists and agonists, there is another type of AR ligand termed selective AR modulators (SARMs). SARMs function as AR agonists in muscle and bone but not in PCa. Therefore, SARMs are considered as potential therapeutics for muscle atrophy and osteoporosis [4], [9]. Currently, a number of SARMs, such as RAD140 [10], MK-4541 [11], and BA321 [12], have been identified using different methods. For instance, RAD140, a nonsteroidal SARM, was obtained through structural modification of an active metabolite of a lead compound [10]. MK-4541 was screened from manually-designed SARMs [9], [11], while BA321 was identified from a group of synthesized carborane derivatives that were expected to interact with hormone receptors [12]. Given their great therapeutic potentials, SARMs such as GTx-024 [13], LGD-4033 [14], and PF-0626414 (NCT02070939) are currently subjected to safety evaluation for potential treatment of muscle wasting and sarcopenia. Nonetheless, the development of SARMs has been limited to traditional experience-based design and chemical synthesis based on known bioactive compounds. Application of VS in SARM studies would help to identify new-scaffold bioactive compounds, and could increase the success rate of SARM development.

Similar to other NRs, AR contains four structural modules, *i.e.*, the N-terminal domain (NTD), DNA binding domain (DBD), hinge, and ligand binding domain (LBD) [15]. Most AR ligands bind to the ligand binding pocket (LBP) in LBD to activate or inhibit the downstream reactions of AR signaling pathway [2]. Therefore, LBD, especially its LBP is a critical target for the identification of novel AR ligands. The marketed AR antagonists (flutamide, nilutamide, Bic, and Enz), newly discovered AR antagonists (VPC-12060 [8], MEL-3 [16], and DIMN [7]), and some SARMs (RAD140 [10] and BA321 [12]) are all designed to bind to AR-LBP. In our current study, we aim to discover novel AR ligands targeting LBP through structure-based VS and bioassays. Based on two identified crystal structures of AR bound with its agonist, we virtually screened the ChemBridge database and obtained 58 AR ligand candidates with new scaffolds. Subsequent bioassays showed that 5 of them exhibited strong binding to AR-LBD and diverse bioactivities. Furthermore, the conformational features of AR-LBD upon the binding of the AR ligands identified in this study were investigated through molecular dynamics (MD) simulations.

## Results and discussion

### Structure-based VS predicts potential AR ligands

**Figure 1:**
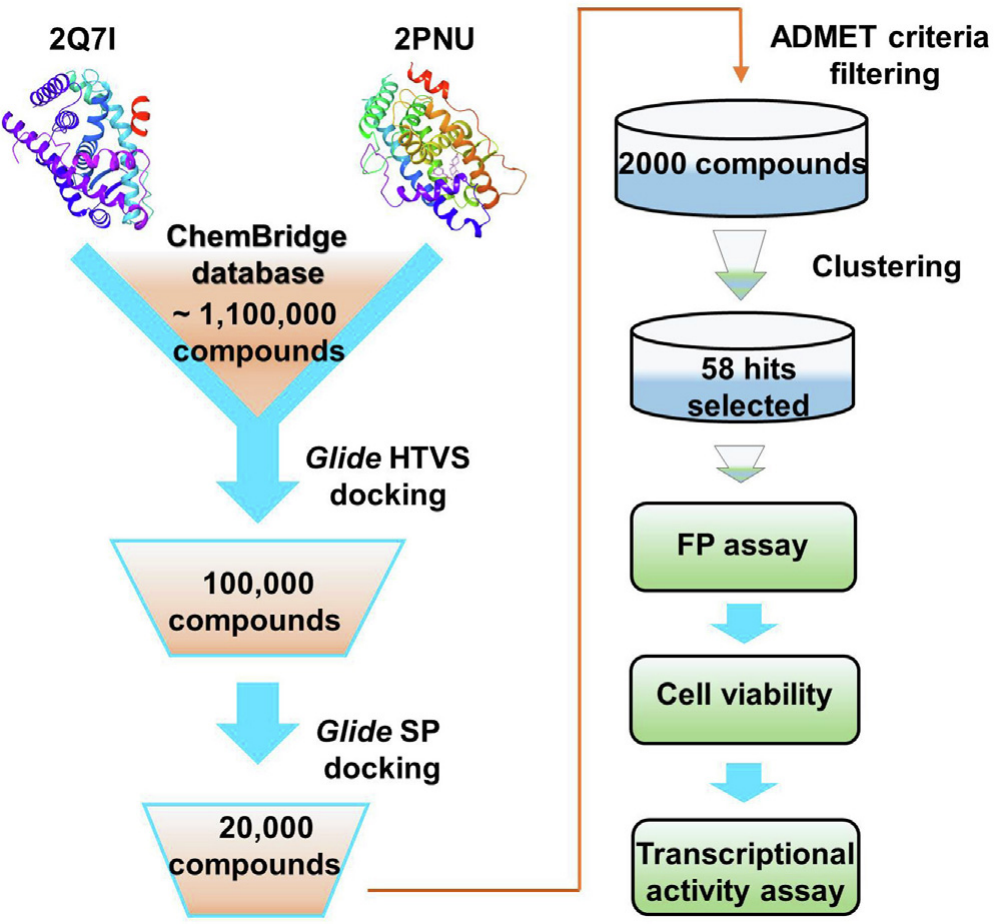
**The workflow of the docking-based virtual screening and bioassay verification** Based on the structures of AR-LBD in complex with testosterone (PDB ID: 2Q71) and EM-5744 (PDB ID: 2PNU), respectively, 58 compounds were finally selected from the ChemBridge database sequentially by *Glide* HTVS docking, *Glide* SP, ADMET criteria, as well as clustering, and then subjected to bioassays. AR-LBD, ligand binding domain of androgen receptor; HTVS, high-throughput virtual screening; SP, standard precision; ADMET, absorption, distribution, metabolism, excretion, and toxicity.

**Supplementary figure 1:**
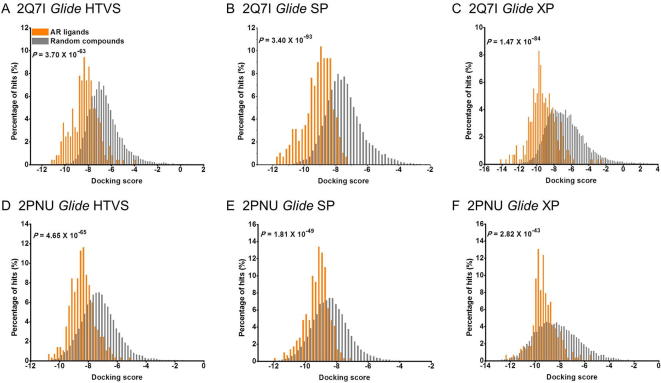


The crystal structures of AR-LBD bound with different ligands exhibit agonistic conformations, making it challenging to identify antagonists through structure-based VS. However, crystal structures of AR-LBD in complex with agonists may serve as the good starting points for the screening of agonists and SARMs. In the present study, two AR-LBD crystal complexes (PDB IDs: 2Q7I and 2PNU) with high resolution (1.87 Å and 1.65 Å) were analyzed to evaluate which crystal structure and which docking mode are more suitable for VS. The root-mean-square deviations (RMSDs) between the docked poses and the original conformations of the ligands in 2Q7I and 2PNU are 0.386 Å and 0.441 Å, respectively, suggesting that the *Glide* docking can successfully reproduce the near-native conformations for the co-crystallized ligands in 2Q7I and 2PNU. Then, the “discrimination power” of molecular docking to discriminate the known ligands from the random decoys was evaluated using the student's *t*-test. As shown in Figure S1, there are visible differences between the two peaks of the distribution of docking score for the AR ligand set and the random compound set in all six docking modes (*P* < 2E − 40). *Glide* docking using the three scoring modes tested was able to efficiently distinguish the known ligands from the decoys. And the SP mode of 2Q7I and high-throughput VS (HTVS) mode of 2PNU showed the best discrimination capacities. To balance calculation time and accuracy, we chose the crystal structure of 2Q7I as the primary template for the *Glide* docking-based VS using the HTVS and then SP scoring modes. After sequential filtration using “Rule-of-Five” [17], the drug-likeness model [18], [19], and the REOS filters [20], and the subsequent clustering, 58 compounds (Table S1) were eventually selected and subjected to subsequent bioassays (Figure 1).

### Competitive binding affinity to AR-LBP

**Figure 2:**
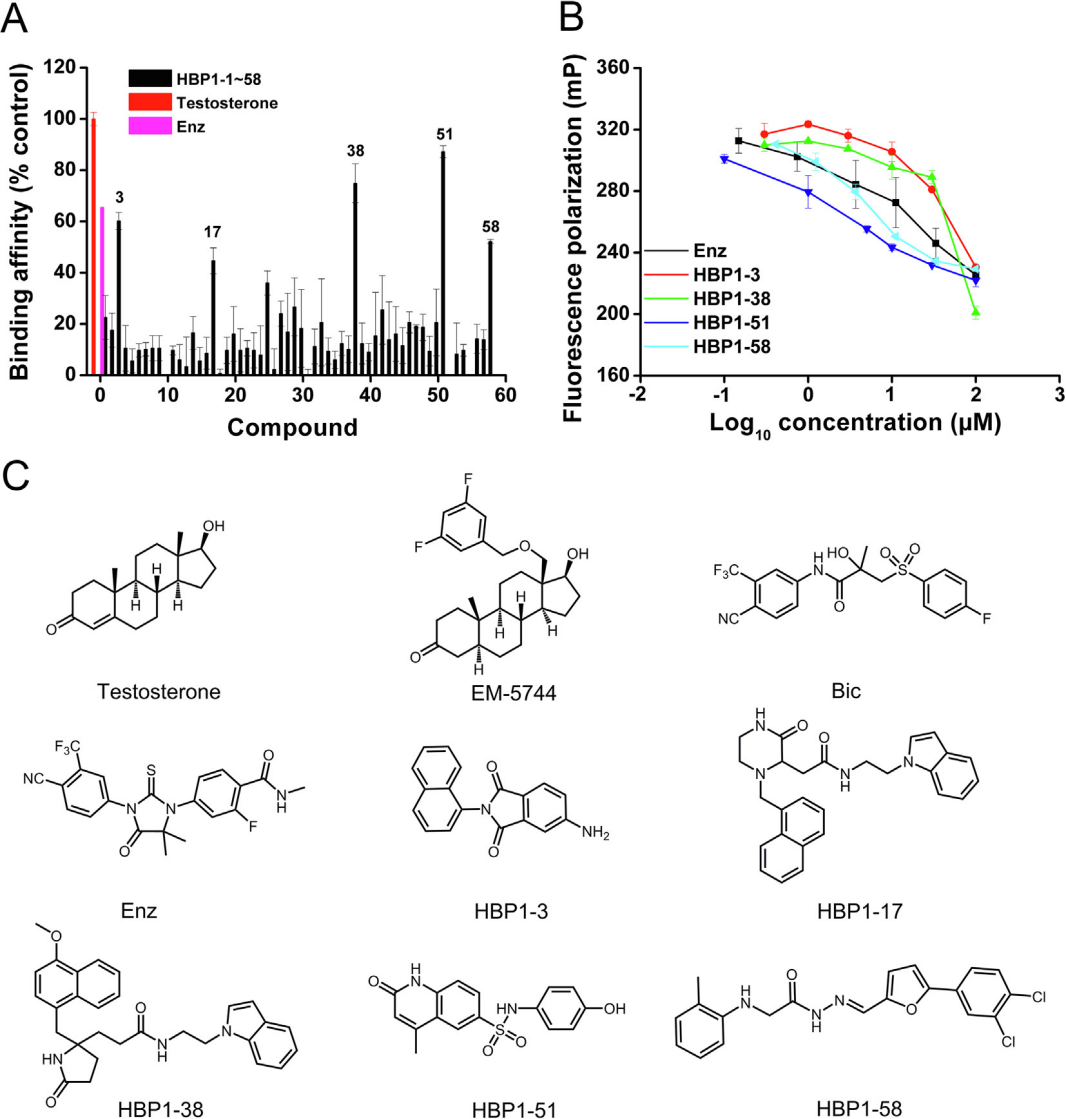
**Competitive binding assay of the 58 compounds identified through VS** **A.** Relative AR binding affinity of the 58 compounds (10 µM) analyzed using PolarScreen™ AR Competitor Assay using DHT as control. The numbers at *X* axis represent the numerical codes of the identified compounds; *e.g.*, 10 represents HBP1-10. The AR binding affinity of DHT was set as 100%. **B.** Four AR favorable compounds HBP1-3, HBP1-38, HBP1-51, and HBP1-58 were evaluated under various concentrations for their precise binding affinity with Enz as control. Standard errors were derived from at least triplicate assays. **C.** Structures of HBP1-3, HBP1-17, HBP1-38, HBP1-51, and HBP1-58, and four known AR ligands tested in this study. These include testosterone (endogenous androgen), EM-5744 (reported AR agonist), Bic (marketed AR antagonist), and Enz (marketed AR antagonist). DHT, dihydrotestosterone; Bic, bicalutamide; Enz, enzalutamide.

To test whether the putative AR ligands identified by VS have high binding affinities to the AR-LBP, we performed the PolarScreen™ AR competitor assay for the selected compounds. The assay was set up comprising AR-LBD and its fluorescence tagged ligand (Flu-AR ligand), whereas addition of a selected compound might disrupt the complex of AR-LBD and Flu-AR by replacing the Flu-AR ligand. The initial binding assay was conducted at 10 µM for all the 58 compounds individually. As shown in Figure 2A, HBP1-3, HBP1-17, HBP1-38, HBP1-51, and HBP1-58 effectively replaced Flu-AR ligand and formed stable complex with AR-LBD. The screening hit rate was 8.6% (5/58). Compared to the natural AR ligand testosterone (binding affinity set as 100% as control), the normalized binding affinity of Enz (also called MDV3100) was 63%, whereas the binding affinities of the five compounds identified ranged from 40% to 90% (Figure 2A). To further analyze the binding affinities of these compounds in detail, we tested their abilities of interrupting the complex of AR-LBD and Flu-AR ligand under gradient concentrations (0.1–100 µM) using Enz as the control. Enz is a marketed second-generation AR antagonist, which exhibits higher binding affinity to LBP than all marketed AR antagonists and many other reported AR ligands [21]. Considering that HBP1-17 and HBP1-38 are analogs, only HBP1-38 was taken for the assay together with HBP1-3, HBP1-51, and HBP1-58. As shown in Figure 2B, all the four compounds tested bound to LBP well in a dose dependent manner. Among them, HBP1-51 (IC_50_ = 3.96 μM) and HBP1-58 (IC_50_ = 4.92 μM) showed higher binding affinity than Enz (IC_50_ = 13.87 μM). The structures of the five identified AR ligands [22] are provided in Figure 2C. We then used the functional-class extended-connectivity fingerprint of length 4 (FCFP_4) fingerprint analysis to compare the 2D molecular structures of these compounds in a binary format with all reported ligands up till now. As shown in Table 1, these compounds had low Tanimoto similarity coefficients (0.21–0.54), suggesting that the identified ligands are not quite structurally similar to any known AR ligands.

**Table 1:** **Docking scores, properties and experimentally-determined bioactivities of the identified AR ligands**

**Compound**	**Binding EC50 (μM)**	**Antagonistic EC50 (μM)**	**Inhibition on cell proliferation (%)**	**Docking score**	**MW**	**Lipid/water partition coefficient (lg)**	**H-bond donor**	**H-bond acceptor**	**Tanimoto similarity coefficient**
Enz	13.87	0.041 ± 0.023	26.3 ± 1.6	−13.18	464.4	2.35	1	6	1
Bic	ND	0.69 ± 0.085	16.5 ± 1.0	−11.60	430.4	2.53	2	6	1
HBP1-3	> 30	NA	7.8 ± 4.4	−11.13	288.3	2.09	2	4	0.36
HBP1-17	> 30	NA	7.8 ± 4.1	−13.66	440.5	3.21	2	6	0.21
HBP1-38	> 30	NA	18.6 ± 4.7	−13.48	469.6	4.51	2	6	0.24
HBP1-51	3.96	Agonist	NA	−11.71	330.4	1.73	3	6	0.54
HBP1-58	4.92	3.25 ± 0.43	2.2 ± 0.8	−11.35	402.3	4.94	2	5	0.27

*Note*: Inhibition on cell proliferation was done in the LNCaP cell line in the presence of the specified compound at 10 μM. The maximal pairwise Tanimoto similarity coefficients for each compound with the 300 AR ligands reported in Binding DB were calculated based on the FCFP_4 fingerprints. MW, molecular weight; Enz, enzalutamide; Bic, bicalutamide; ND, not determined; NA, not applicable.

### Bioactivities of the selected AR ligands for prostate cancer cells

**Figure 3:**
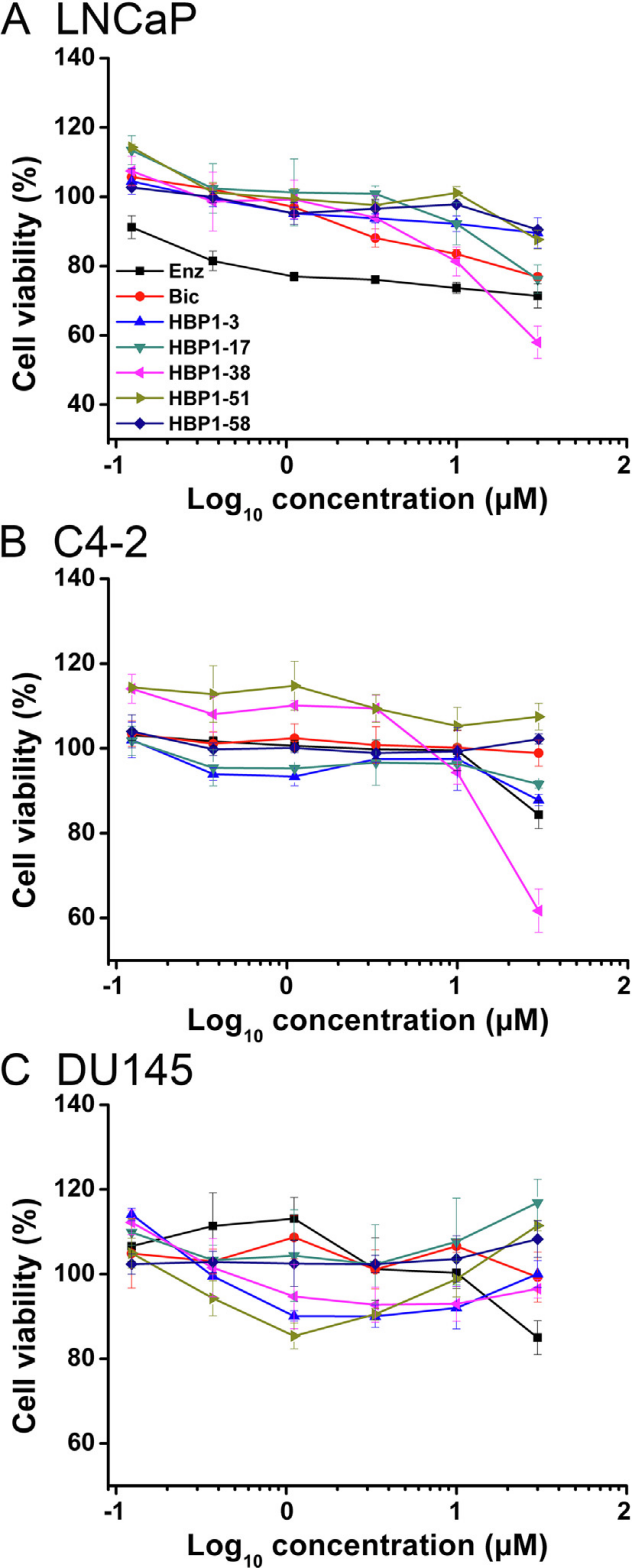
**Cell viability assay of the selected AR ligands** Five AR ligands HBP1-3, HBP1-17, HBP1-38, HBP1-51, and HBP1-58 were analyzed with various prostate cancer cell lines including LNCaP (**A**), C4-2 (**B**), and DU145 (**C**). Enz and Bic were taken as controls. The cell viability assay was repeated at least 3 times for each compound at each concentration. Viability of cells without treatment of compound was set as 100%.

We then asked the question whether the five identified AR ligands are able to affect the proliferation of AR-related cell lines, especially LNCaP, the androgen dependent AR^+^ PCa cells. To answer this question, we cultured the LNCaP cell line with the tested compounds at various concentrations, using Bic/Enz as the control. As shown in Figure 3A, Bic and Enz both reduced the growth of LNCaP cells in a dose-dependent manner. Nevertheless, we only observed modest inhibition of cell growth when the screened compounds were applied at concentrations below 10 µM. At 10 µM, HBP1-38 showed inhibitory effect comparable to Bic. Compared to the cells not treated by compounds, a significant decrease in cell viability was observed when cells were treated with HBP1-3, HBP1-17 (≥20%), HBP1-38 (≥40%), and HBP1-58 (all with *P* = 0.0001; 1-way ANOVA) at 30 µM. Overall, HBP1-51 did not show apparent inhibition tendency for the growth of LNCaP. These data suggest that except for HBP1-51, the compounds with strong AR-LBD affinity tend to function as AR antagonists in LNCaP cells.

We further tested C4-2, an androgen-independent AR^+^ PCa cell line, which is more malignant than LNCaP. As shown in Figure 3B, none of the compounds including Bic appeared to inhibit the cell proliferation at concentration below 10 µM. Conversely, different from the other examined compounds, HBP1-51 at all tested concentrations increased the viability of C4-2 cells compared to the untreated cells, suggesting that HBP1-51 behaves like an agonist, although the activating effect is not significant (*P* = 0.14; 1-way ANOVA) with the maximum increase below 20%. At 30 µM, the cell viability of C4-2 was significantly reduced nearly 40% by HBP1-38, 12% by HBP1-3, and 10% by HBP1-17 (all with *P* = 0.0001; 1-way ANOVA), compared to cells without treatment by compounds. Given inhibitory effect was only observed at higher concentrations, it remains to be addressed whether the inhibition is attributed to AR blocking or the inherent toxicities of the tested compounds. We thus tested these compounds on DU145, an AR^−^ PCa cell line. As shown in Figure 3C, the cell viability curves of DU145 showed a smile shape for all the five tested compounds at varied concentrations ranging from 0.1 µM to 30 µM, thus ruling out the possibility of cellular toxicities. In summary, except for HBP1-51, the other four compounds tested all exhibit inhibitory effect on the growth of AR^+^ PCa cells, although at various degrees.

### Regulation of AR transcriptional activities by the selected AR ligands

**Figure 4:**
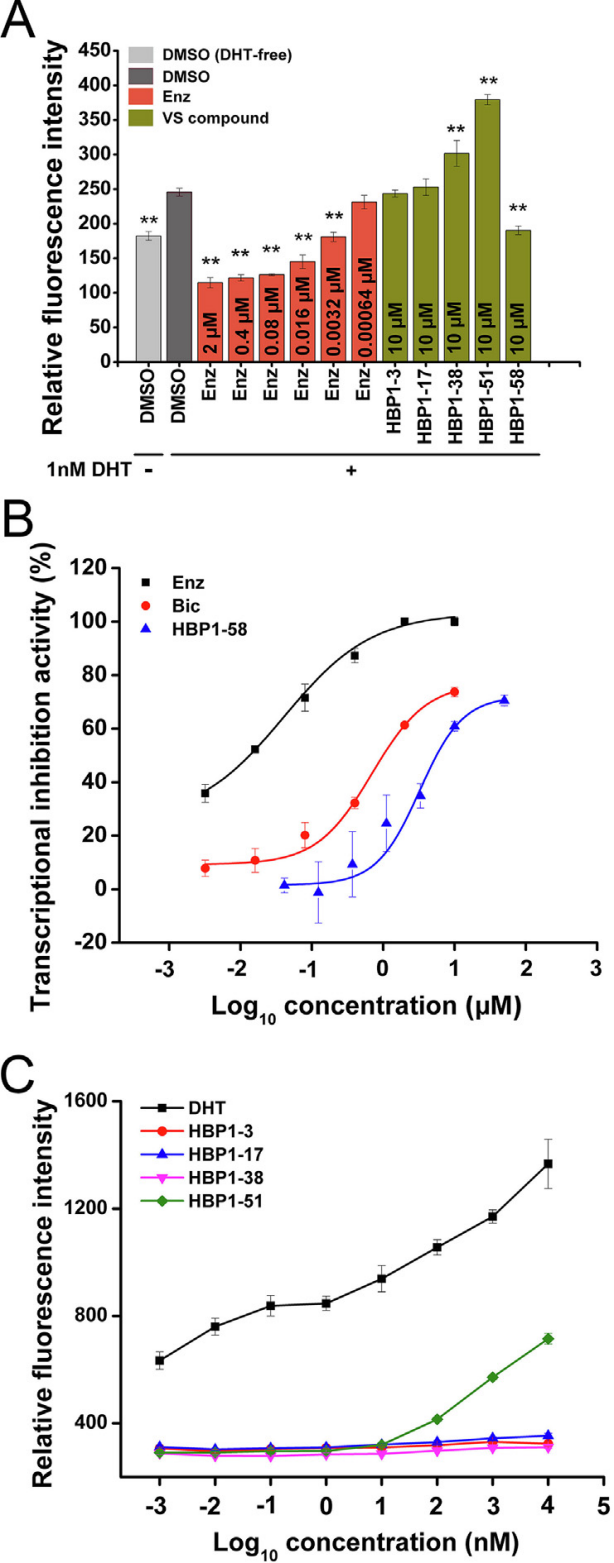
**The efficacy of the selected AR ligands on transcriptional activity assay of AR** **A.** Compounds HBP1-3, HBP1-17, HBP1-38, HBP1-51, and HBP1-58 were subjected to LNCaP-ARR_2_PB-eGFP based transcriptional activity assay to assess their antagonist activities. Enz was used as a control and incubated with cells at concentrations from 0.0032 µM to 2 µM, while the five tested compounds were incubated with the cells at 10 µM. 1 nM DHT was added for background fluorescence. In addition, cells receiving equal volume of DMSO solution with (dark gray bar) or without (light gray bar) DHT served as controls too. **B.** Transcriptional activity assay of HBP1-58, using Enz and Bic as positive controls. **C.** Androgen like activities of compounds HBP1-3, HBP1-17, HBP1-38, and HBP1-51 were evaluated using LNCaP-ARR2PB-eGFP based transcriptional activity assay without preincubation of DHT. The test concentrations range from 0.001 nM to 10 µM. DHT served as the positive control. Assays were performed in triplicate. Multiple group comparisons were analyzed with 1-way ANOVA (**, *P* < 0.05 were considered statistically significant).

Since AR ligands could serve as agonists, antagonists, or SARMs, it is important to examine how the identified AR ligands function when they bind to AR. For this purpose, we established a stable cell line of LNCaP-ARR_2_PB-eGFP in which the expression of enhanced green fluorescence protein (eGFP) is controlled by the AR-response element of the engineered ARR_2_PB sequence. ARR_2_PB carries an extraordinarily strong promoter with relatively short sequence, and can be specifically triggered by activated AR [23]. Therefore, the expression level of eGFP in LNCaP-ARR_2_PB-eGFP is strongly dependent on the intensity of AR activation. Such method has been employed to quantitatively assess AR ligands [24]. In the current study, the LNCaP-ARR_2_PB-eGFP cells were cultured with 10 µM tested compounds individually in the presence of DHT. Enz was added at concentrations ranging from 6.4 × 10^−4^ µM to 2 µM as the control. As shown in Figure 4A, the addition of DHT (dark gray bar) significantly (*P* = 0.0001; 1-way ANOVA) upregulated the expression level of eGFP compared to the cells treated with DMSO alone (light gray bar). Presence of 0.0032 µM Enz was sufficient to cause significant downregulation (*P* = 0.0001; 1-way ANOVA) of eGFP expression compared to the control (dark gray bar). As for the tested compounds, HBP1-58 significantly reduced eGFP expression, whereas HBP1-38 and HBP1-51 significantly (*P* = 0.0001; 1-way ANOVA) promoted eGFP expression in the presence of DHT, seemingly playing similar role as DHT. HBP1-3 and HBP1-17 did not show obvious effect on eGFP expression. These data indicate that HBP1-3, HBP1-17, HBP1-38, and HBP1-51 are most likely not AR antagonist candidates. Further assays were performed for HBP1-58 to verify its antagonistic effects. The compound exhibited outstanding inhibition effect with IC_50_ = 3.25 ± 0.43 µM (Figure 4B).

The agonistic effects of the screened compounds were analyzed by stimulating the cell growth of LNCaP-ARR_2_PB-eGFP without prior addition of DHT. Cells were incubated with various concentrations of HBP1-3, HBP1-17, HBP1-38, and HBP1-51, respectively, ranging from 1 × 10^−3^ nM to 1 × 10^4^ nM (Figure 4C). As expected, DHT upregulated eGFP expression in a dose-dependent manner, whereas HBP1-3, HBP1-17, or HBP1-38 did not cause notable change at the tested concentrations. Interestingly, a dose-dependent upregulation of GFP expression was observed in the presence of HBP1-51 at concentrations over 1 × 10^2^ nM, although the upregulation was much lower than that for DHT. These findings are consistent with the observations from the previous cell proliferation assays, indicating that HBP1-51 is a relatively weak AR agonist. It seems that HBP1-38 could activate the AR promoter cooperatively with DHT, as the transcriptional activity with both HBP1-38 and DHT (Figure 4A) was higher than that with DHT alone (Figure 4A, dark gray bar). On the other hand, considering their bioactivity profiles, *i.e.*, favorable LBP binding affinities and moderate inhibition on the proliferation of AR^+^ PCa cells, just like reported for SARMs, we speculate that HBP1-3, HBP1-17, and HBP1-38 may regulate AR function similarly as SARMs.

### The predicted binding poses of the identified ligands

**Figure 5:**
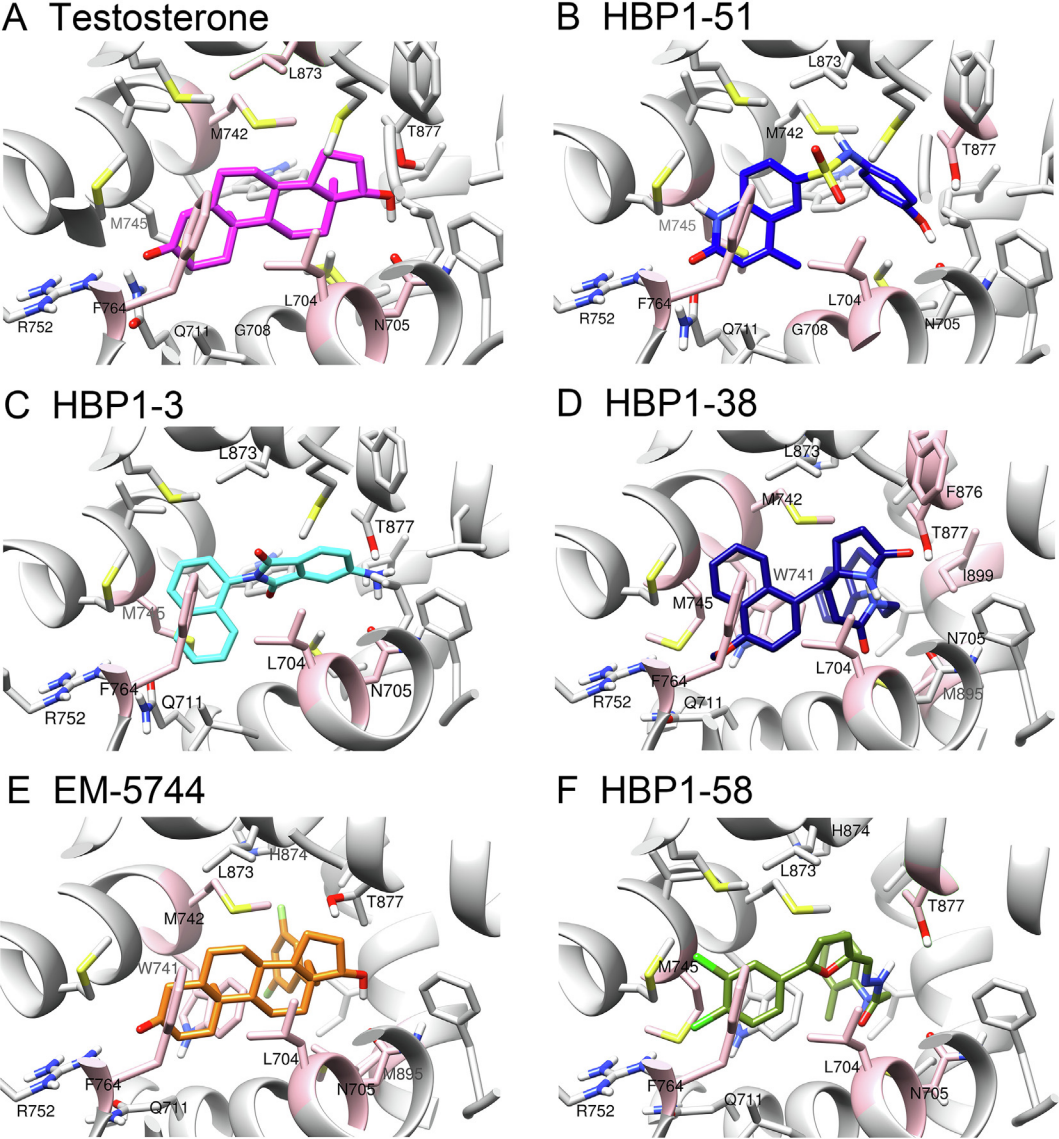
**The binding poses of the AR ligands in the AR-LBD** The binding poses of testosterone (**A**), HBP1-51 (**B**), HBP1-3 (**C**), HBP1-38 (**D**), EM-5744 (**E**), and HBP1-58 (**F**) in the binding pocket of AR-LBD were predicted using molecular docking. The binding poses of testosterone and EM-5744 were directly captured from the crystal structures, and poses of the four HBP ligands were generated using the *Glide* docking. Residues identified by the molecular mechanics/generalized born surface area (MM/GBSA) free energy decomposition were highlighted in pink.

To demonstrate the receptor-ligand interaction patterns, we analyzed the docking poses generated by *Glide* SP scoring. We found that the binding mode of HBP1-51 is highly similar to that of the steroid androgen in the crystal structure of AR/testosterone complex (PDB ID: 2Q7I) (Figure 5A and B). The majority of both ligands adopted nearly the same orientation, and the plane of the three discontinuous rings in HBP1-51 spreads out in parallel, which is quite similar to the steroid skeleton of androgen. We also found that the carbonyl group of HBP1-51 is located roughly at the same site as the 3-keto group of testosterone, and the distances between the polar hydrogen atom of Arg752 and the carbonyl oxygen atoms of HBP1-51 and testosterone are 1.65 Å and 2.27 Å, respectively. In addition, the hydroxyl of the phenol ring in HBP1-51 occupies the position where the hydroxyl of the D ring in the steroid ligand is located, with a distance of 1.7 Å away from the side chain hydrogen bond receptor of Gln705 and ∼2.6 Å away from the side chain hydroxyl of Thr877. Therefore, both the polar oxygen atoms at the two opposite ends of the ligands can form three similar hydrogen bonds with the side chains of Arg752, Asn705, and Thr877. In addition, both ligands are encircled within a hydrophobic cylinder formed by several non-polar residues including Leu704, Met742, Met745, Phe764, and Leu873. The higher binding affinity and stronger androgenic effect of testosterone over HBP1-51 may be explained by the more favorable interactions between the aliphatic steroid rings and the hydrophobic cylinder [25].

The docking results could provide some insights into the possible reason explaining the lower binding affinities of HBP1-3 and HBP1-38 to AR than HBP1-51. According to the predicted binding pose (Figure 5C), the backbone of HBP1-3 exhibits moderate similarity to that of HBP1-51. The primary amino group of HBP1-3 almost occupies the same space as the hydroxyl group of HBP1-51 and accordingly forms two hydrogen bonds with the side chains of Asn705 and Thr877. Nevertheless, absence of hydrogen bond donor or acceptor on the naphthyl ring leads to weaker electrostatic interactions with the polar amino acids of Gln711 and Arg752 on the other end. Besides, the two oxygen atoms at both sides of 1,3-dione are surrounded by hydrophobic residues, which may impair its binding capacity with AR-LBD as well. Similarly, compared to the carbonyl oxygen atom in HBP1-3 or the steroid ring, the oxygen atom of HBP1-38 in the 4-methoxynaphthalen group cannot form hydrogen bonds with Gln711 and Arg752, but is encompassed by the hydrophobic residues of Met749, Phe764, and Met787 instead.

In contrast to these two weak binders, the antagonistic ligand HBP1-58 exhibits stronger binding as indicated by the predicted pose. One of the chlorine atoms could serve as a hydrogen bond acceptor and forms an H-halogen interaction with Arg752, whereas the two polar hydrogen atoms located in the amide group and secondary amine group of HBP1-58 could form hydrogen bond contacts with the side chain of Asn705. Unlike the bulky naphthalene ring that produces adverse steric hindrance nearby helix 5 in HBP1-3 and HBP1-38, the dichloro-substituted phenyl ring in HBP1-58 may form stronger van der Waals and hydrophobic interactions with the side chains of Met745, Met749, and Phe764. This may confer the ligand higher binding affinity with AR, and this phenomenon could be validated by these antagonists used clinically.

### The dynamic interaction patterns of the novel ligands identified

To generate an ensemble view of the dynamic behaviors of our screened ligands to AR, we evaluated the AR/ligand complexes using MD simulations. As shown in Figure S2A, the RMSDs of the backbone C_α_ atoms tended to become stable after about 10 ns and all the simulated systems achieved equilibrium during the last 20 ns. The average mobility of the AR-LBD backbone was then characterized using the root mean square fluctuations (RMSFs) of the C_α_ atoms (Figure S2B). As expected, majority of AR-LBD in complexes with different ligands displayed highly similar patterns. However, RMSFs of helix 12 in the complexes formed between AR and antagonists (Enz and HBP1-58) were obviously larger than those in the complexes formed between AR and agonists (testosterone and HBP1-51), suggesting that compared to agonist, binding of antagonist would enhance the mobility of helix 12 of AR-LBD as illustrated in Figure S2B.

**Figure 6:**
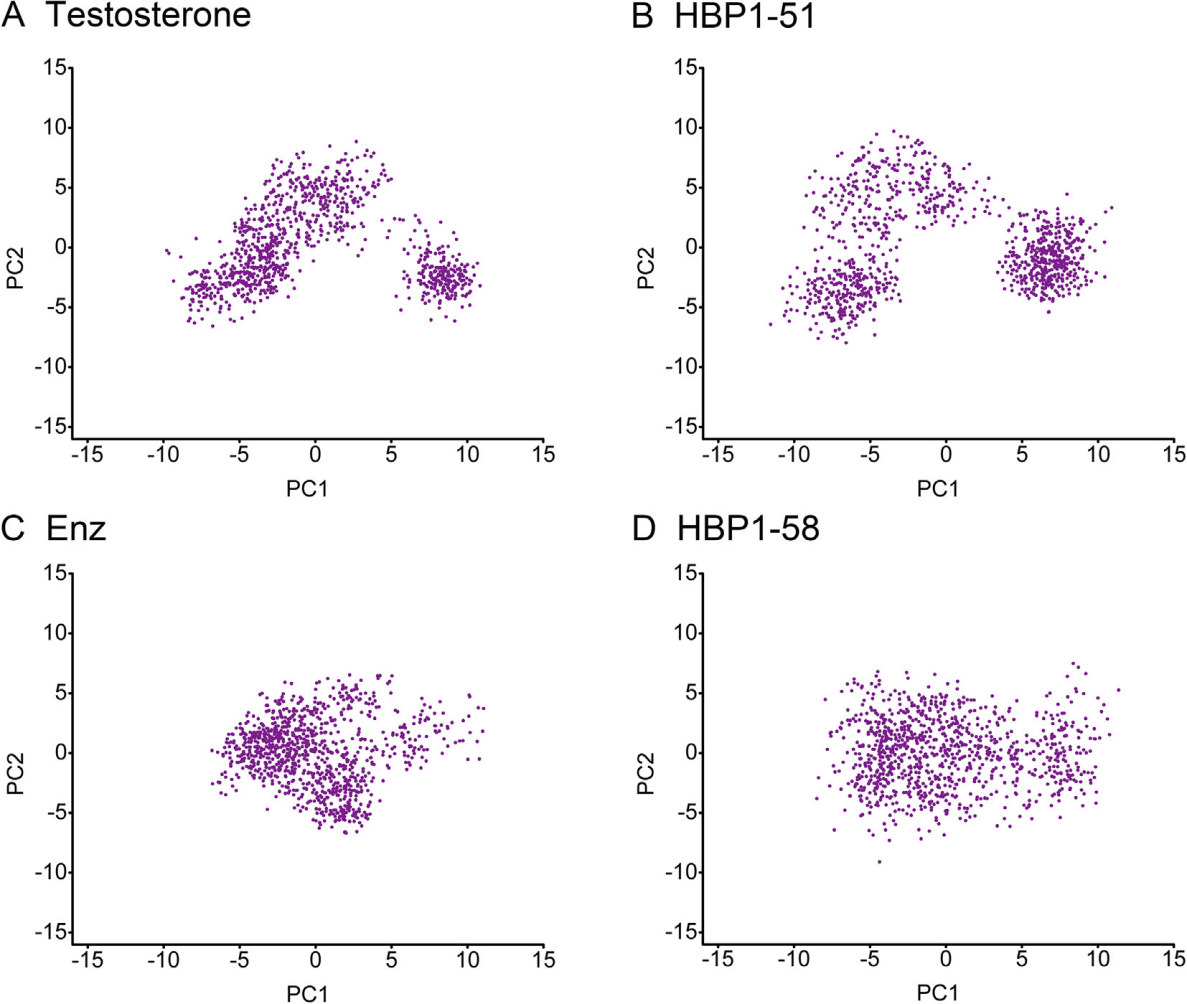
**Principal components analysis of the Cα atoms of the AR conformational motions during simulation** The cloud represents the last 10 ns of trajectories projected onto the first two eigenvectors for the AR/testosterone (**A**), AR/HBP1-51 (**B**), AR/Enz (**C**), and AR/HBP1-58 (**D**). The distribution of dots indicates the conformational changes in the system, with each dot representing a snapshot from MD simulation trajectory. MD, molecular dynamics.

To further analyze the conformational change of AR induced by ligand binding, we examined the conformational distribution of AR bound with agonists or antagonists using PCA. As shown in Figure 6, the clouds represent the conformational changes in the simulated systems, with each dot representing a snapshot from MD simulation trajectory. It can be observed that the overall conformational changes of AR/testosterone and AR/HBP1-51 are similar, while those of AR/Enz and AR/HBP1-58 are similar. These data indicate that HBP1-51 and testosterone induce similar conformational changes of AR, while HBP1-58 induces conformational changes of AR similar as Enz.

**Figure 7:**
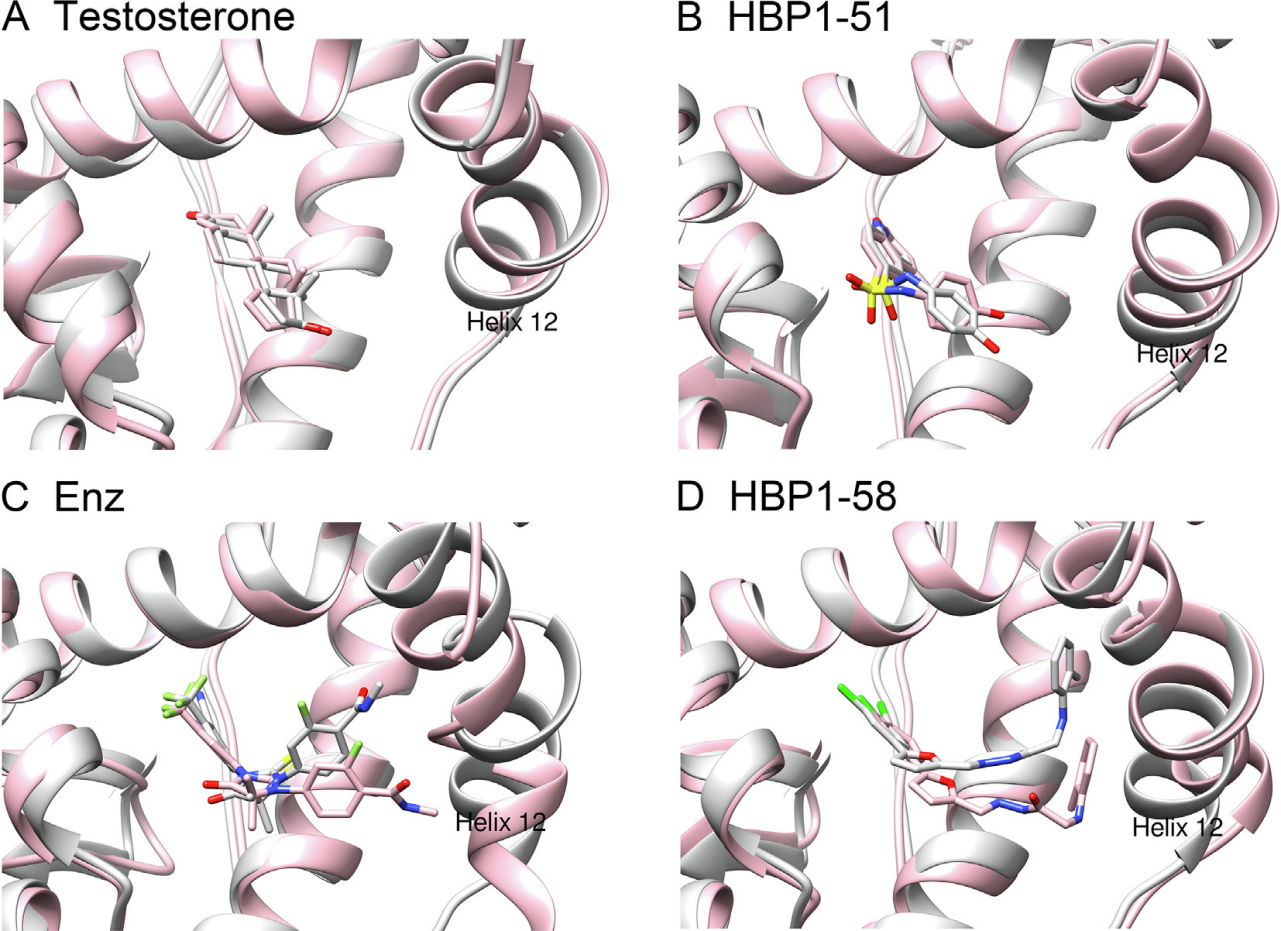
**The agonistic and antagonistic state of AR-LBD complexed with the selected AR ligands revealed by MD simulations** Shown are the conformational changes of helix 12 after 30 ns MD simulations for testosterone (**A**), HBP1-51 (**B**), Enz (**C**), and HBP1-58 (**D**). The initial structures of the receptor/ligand complexes were colored in light gray, and the conformations produced by the MD simulations were colored in pink.

Previous studies suggest that antagonists but not agonists would induce repositioning of helix 12 of AR-LBD. Therefore, we performed the structural analysis to examine the conformational change of the helix 12 induced by the screened ligands [26], [27]. The structural alignments of the first snapshots and last snapshots extracted from the MD trajectories showed that helix 12 maintained its position tightly when the agonist testosterone (Figure 7A) or HBP1-51 (Figure 7B) occupied the active site. However, helix 12 had an obvious conformational movement when the antagonist Enz (Figure 7C) or HBP1-58 (Figure 7D) bound to the active site. These simulation studies highlight the conformational change of helix 12 induced by the binding of an antagonist, which may disturb the binding of coactivators and impair the following functions of AR.

Overall, our simulations are consistent with the previous studies showing that the binding of an agonist would enhance the stability of the tertiary structure of AR, while the binding of antagonist may damage the harmonious “sandwich” structure and accordingly perhaps lead to the failure of crystallization [28].

## Conclusion

In this study, we have applied a structure-based VS to search for novel AR ligands and identified 58 compounds for bioassay validation. Among them, five novel AR ligands with strong AR-LBD affinities, including HBP1-3, HBP1-17, HBP1-38, HBP1-51, and HBP1-58, were identified. HBP1-51 and HBP1-58 exhibit stronger androgen displacement ability than Enz, a second-generation AR antagonist. Further bioassays indicate HBP1-51 as a relatively weak AR agonist and HBP1-58 as an AR antagonist. HBP1-3, HBP1-17 and HBP1-38 exhibit SARM-like bioactivity profile in our assays. We speculate that these three compounds might function as SARMs, but further validation studies using animal models are needed before a solid conclusion can be reached. Furthermore, our MD simulation analysis suggests that the binding by an antagonist or an agonist exerts quite different impacts on the conformational change of helix 12, thus providing valuable information for the design of novel and specific antagonists or agonists targeting AR-LBD. More importantly, the new-scaffold AR ligands identified in this study could serve as a good starting point for further medicinal chemical studies such as structural modification, to facilitate the development of new drug candidates for PCa, muscle atrophy, osteoporosis, *etc*.

## Materials and methods

### Preparation of crystal complexes and validation dataset for docking-based VS

The crystallographic structures of the human wild type AR-LBD complexed with agonists EM5744 (PDB ID: 2PNU) and testosterone (PDB ID: 2Q7I) [29] for molecular docking were downloaded and prepared by the *Protein Preparation Wizard* module in Schrödinger 2015 (Schrödinger, LLC, New York, NY). All crystallographic ions, water, and dithiothreitol (DTT) molecules were removed, missing protein atoms were added, protonated states and partial charges were assigned, and the structure was then minimized. The validation dataset was prepared similarly as previously reported [30], [31] and used to evaluate the prediction capacity of *Glide* based on the two crystal structures. The known non-steroid AR ligands with experimental bioactivities (*K*_i_ < 10 μM) were retrieved from the BindingDB database [22]. 300 diverse AR ligands were selected based on the similarity represented by Tanimoto coefficient calculated using the FCFP_6 fingerprints (Accelrys Inc, San Diego, CA). Since the chemical space of a decoy set could be well represented by a set of compounds randomly selected from a commercial database [32], the AR non-ligands in the validation dataset were selected randomly from the ChemBridge database. Considering that the non-ligands *versus* the known ligands of AR is quite unbalanced, the ratio between the numbers of non-ligands and ligands in the validation dataset was set to 60. For the molecules in the validation dataset, the *Epik* module was used to generate the ionized states and tautomers/stereoisomers at pH = 7.0 ± 2.0 (Schrödinger, LLC, New York, NY).

### Docking-based VS

Prior to screening the ChemBridge database, the performance of *Glide* based on the two crystal structures (2Q7I and 2PNU) was evaluated. For each complex, the binding pocket was defined to the region centered on the mass center of the co-crystallized ligand with the size of 10 Å × 10 Å × 10 Å using the *Receptor Grid Generation* component of *Glide* [33]. Testosterone and EM5744 were first extracted and then re-docked into the crystal structures. Then, the compounds in the validation dataset were docked using three scoring functions, including HTVS, SP and XP (extra precision). Finally, the performances of *Glide* for two different AR structures and three scoring modes were evaluated.

The ChemBridge database contains about 1.1 million compounds and was used as the screening library. Compounds in ChemBridge were processed using the *LigPrep* mode in Schrödinger 9.0 (Schrödinger LLC, New York, NY). All the prepared small compounds were initially docked by *Glide* with the HTVS scoring, and the 100,000 top-ranked structures were then re-docked and rescored using the *Glide* SP scoring system. The non-duplicated top-ranked 20,000 compounds were then filtered according to “Rule-of-Five” [17], the drug-likeness model [18], [19], and the rapid elimination of swill (REOS) rules [20]. After structural clustering, 58 compounds were selected and then purchased from Target Molecule Corp (Boston, MA) for further bioassays.

### AR binding assay

Fluorescence polarization assay was employed to assess the binding affinity of the identified compounds to AR-LBD through PolarScreen™ Androgen Receptor Competitor Assay Kit (Thermo Fisher Scientific, Waltham, MA). Briefly, 10 μl 2× test compound (or DHT, Enz, DMSO) was dispersed in low-volume 384-well plate (Corning, Cat. No. 4514), followed by adding 4× AR-LBD (GST) and 4× Fluormone AL Green dissolved in complete AR Green Assay Buffer. The assay plate was stirred moderately to mix the ingredients and covered with aluminized paper to protect reagents from light. After the plate was incubated at room temperature for 5 h, the fluorescence polarization value (mP) of each well was measured on a multi-function plate reader (Synergy H1, BioTek, Winooski, VT).

### Transcriptional activity assay

A cell line of LNCaP that stably expresses eGFP under the regulation of an androgen response element was generated to investigate the agonist/antagonist activity of the selected compounds as previously described [24], [34]. Firstly, the CMV promoter in the transfer plasmid pLJM1-eGFP (#19319, Addgene) was removed and replaced with the androgen induced ARR_2_PB promoter. The lentiviral vector was co-transfected with 10 μg of pLJM1-ARR_2_PB-eGFP plasmids, 10 μg of pCMV-dR8.2 (#8455, Addgene), and 5 μg of pMD.RVG.CV24-B2c (#19713, Addgene) in 293 T cells using the GM easy™ Lentiviral Packaging Kit (Genomeditech, Cat. No. GMeasy-10, Shanghai, China) the day after cell seeding. Two days later, the viral particles in the medium were collected by filtrating through a 0.45 μM filter and then stored at −80° for future use. For lentiviral infection, LNCaP cells were plated and proliferated to a confluence of 60% before adding 5 ml of the collected lentivirus. Medium was refreshed 16 h later and cells were incubated for another two days. GFP expression in the transfected cell was examined with a fluorescent microscope. Subsequently, the infected cells were cultured in complete medium containing 1 μg/ml puromycin. This procedure was repeated for three cycles in the next subculture process to adequately eliminate the non-transfected clones. Cells surviving the screening process were AR-regulated eGFP-expressing LNCaP cells (LNCaP-ARR2PB-eGFP) and were cultured for subsequent antagonist/agonist assay.

### Cell proliferation assay

3-(4,5-Dimethythiazol-2-yl)-2,5-diphenyl-2-H-tetrazolium bromid (MTT) colorimetric assay was utilized to evaluate cell proliferation, which can reflect the bioactivity and cytotoxicity of test compounds at cellular level. PCa cells were cultured in 1640 RPMI media containing 5% charcoal-stripped serum (CSS) at a density of 5000 cells per well for LNCaP, 1000 cells per well for C4-2, and 2000 cells per well for DU145 in 96-well plates. After incubation at 37 °C for 24 h, cells were treated with serial dilutions of indicated compounds (and 1 nM DHT for LNCaP culture) and incubated for another 3 days. Afterward, 10 μl of 5 mg/ml MTT solution was added into each well and further incubated for another 4 h. Then, 100 μl of triplex 10% SDS-0.1% HCl-PBS solutions were added to dissolve the formazan deposited on the bottom of the plates and the plates were then further retained in incubator overnight. The absorbance at 570 nm was measured with the reference wavelength at 650 nm with a spectrophotometer (Bioteck Eon, Winooski, VT).

### Molecular dynamics simulation

The dynamic binding patterns of agonist/antagonist with AR-LBD were examined by MD simulation. The complexes AR/testosterone, AR/HBP1-51, AR/Enz, and AR/HBP1-58 predicted by molecular docking were submitted to the MD simulation. The atomic partial charges for each AR ligand were obtained by fitting the electrostatic potential computed at Hartree-Fock (HF) SCF/6-31G* basis supported in Gaussian09 (Gaussian Inc, Wallingford, CT) using the restrained electrostatic potential (RESP) algorithm [35]. The force field parameter files of the ligands were generated using the *antechamber* module in AMBER14 [36]. The gaff and ff99SB force fields were assigned to the ligands and proteins, respectively [37], [38]. Each complex was solvated into a cubic transferable intermolecular potential with 3 points (TIP3P) water box with 12 Å away from the surface of the protein complex, and 2 Cl^−^ ions were added to neutralize the system. The particle mesh Ewald (PME) algorithm was used to compute the long-range electrostatics [39].

A four-step minimization protocol was used to relax each system. (1) only the hydrogen atoms were optimized by 4000 steps of steepest descent and 1000 steps of conjugate gradient minimizations; (2) the water molecules and counter ions were optimized by 4000 steps of steepest descent and 1000 steps of conjugate gradient minimizations; (3) the side chains the protein and the solvent molecules were optimized by 4000 steps of steepest descent and 1000 steps of conjugate gradient minimizations; (4) the whole system was optimized for 5000 steps of steepest descent and 5000 steps of conjugate gradient minimizations.

Conventional MD simulations were conducted using the *pmemd* module in AMBER 14 [36]. Each system was gradually heated to 300 K over a period of 50 ps and then equilibrated over 20 ps in the NPT ensemble (temperature = 300 K and pressure = 1 atm), before being submitted to 30 ns NPT (temperature = 300 K and pressure = 1 atm) MD simulations. Covalent bonds involving hydrogen atoms were constrained by the SHAKE method [40] with the time set to 2 fs.

### Principal component analysis

PCA is an important method to investigate the collective dynamics of a protein. Here, the distributions of the AR conformations were analyzed using the last 10 ns trajectories based on the following covariance matrix (Equation 1):

(1) $$\sigma_{\mathrm{ij}}=<\left(r_{i}-<r_{i}>\right)\left(r_{j}-<r_{j}>\right)>$$

where *r*_1_, *r*_2_, … , *r*_3N_ represent mass-weighted Cartesian coordinates and < … > is the ensemble average. The symmetrical matrix *σ*_ij_ can be diagonalized to obtain eigenvectors (PC1, PC2, … , PCn) and eigenvalues (*λ*_1_, *λ*_2_,…, *λ*_n_). Here, PC1 and PC2 are sufficient to characterize the conformational distribution for each system.

### Statistical analysis

The statistical differences between groups were determined using Student’s t-test or one-way analysis of variance (1-way ANOVA) for multiple comparisons in GraphPad Prism 7.0 (GraphPad, San Diego, CA). Data were presented as mean ± SD. Differences were considered to be significant at *P* < 0.05.

## Authors’ contributions

TH and DL conceived the idea and supervised the study. WZ, MD, and WF conducted the virtual screening and MD simulations. WZ, JP, and QT conducted the bioassays. WZ, HS, LX, and SC analyzed the data. WZ drafted the manuscript. TH and DL edited the manuscript. All authors read and approved the final manuscript.

## Competing interests

The authors declare no competing interests.

## Figures and Tables

**Supplementary figure 2:**